# Effects of Dietary Components on Mast Cells: Possible Use as Nutraceuticals for Allergies?

**DOI:** 10.3390/cells12222602

**Published:** 2023-11-10

**Authors:** Sina Kaag, Axel Lorentz

**Affiliations:** Institute of Nutritional Medicine, University of Hohenheim, D-70593 Stuttgart, Germany

**Keywords:** allergy, mast cells, nutraceuticals

## Abstract

Allergic diseases affect an estimated 30 percent of the world’s population. Mast cells (MC) are the key effector cells of allergic reactions by releasing pro-inflammatory mediators such as histamine, lipid mediators, and cytokines/chemokines. Components of the daily diet, including certain fatty acids, amino acids, and vitamins, as well as secondary plant components, may have effects on MC and thus may be of interest as nutraceuticals for the prevention and treatment of allergies. This review summarizes the anti-inflammatory effects of dietary components on MC, including the signaling pathways involved, in in vitro and in vivo models. Butyrate, calcitriol, kaempferol, quercetin, luteolin, resveratrol, curcumin, and cinnamon extract were the most effective in suppressing the release of preformed and de novo synthesized mediators from MC or in animal models. In randomized controlled trials (RCT), vitamin D, quercetin, *O*-methylated epigallocatechin gallate (EGCG), resveratrol, curcumin, and cinnamon extract improved symptoms of allergic rhinitis (AR) and reduced the number of inflammatory cells in patients. However, strategies to overcome the poor bioavailability of these nutrients are an important part of current research.

## 1. Introduction

It is estimated that more than 30% of the world’s population is affected by one or more allergic diseases. And the prevalence is increasing worldwide [1,2]. The most common allergies worldwide include allergies to pollen, house dust mites, animal dander, and certain food allergens [3,4]. Their high prevalence may be due to a combination of genetic and environmental factors. Exposure to allergens, pollutants, and changes in living conditions can affect a person’s immune response [5,6,7].

In general, allergy is an overreaction of the immune system to a normally harmless substance, usually a protein, which manifests itself as a skin rash, sneezing, swelling of the mucous membranes, or other abnormal conditions [8]. The term “allergy” was first coined in the early 1900s by Clemens von Pirquet (1874–1929), who was the first to recognize that antibodies not only mediate protective immune responses but can also be the cause of hypersensitivity reactions [9]. Mast cells (MC) are part of the innate immune system and are the key effector cells of type 1 hypersensitivity allergic reactions as well as of other inflammatory diseases [10]. Their activation can be induced by various stimuli, including immunoglobulin (Ig) E-dependent or IgE-independent immune mechanisms. In response to a specific allergen, IgE antibodies bind to the high-affinity FcεRI receptor on the surface of MC, thus leading to their activation by allergen-induced cross-linking [10]. In addition, MC express the Mas-related G protein-coupled receptor type 2 (MRGPRX2), which can be activated by small molecules such as compound 48/80 (C48/80) or substance P (SP) and has been implicated in pseudo-allergic reactions or IgE-independent allergic reactions [11,12]. MC can also be activated by Toll-like receptors (TLR) that recognize pathogen-associated molecular patterns (PAMP) or by exposure to certain cytokines such as interleukin (IL)-33, thymic stromal lymphopoietin (TSLP), and interferon-gamma (IFN-γ) [13]. 

MC promote inflammation by releasing a wide range of inflammatory mediators upon activation. These include pre-stored mediators in MC granules, such as histamine and proteases, as well as de novo synthesized mediators such as cytokines and eicosanoids [14]. The transcription factor nuclear factor kappa B (NF-κB) is an important mediator of inflammation and regulates the expression of pro-inflammatory cytokines [15]. In addition, other signaling molecules such as mitogen-activated protein kinases (MAPK) or signal transducer and activator of transcription (STAT) also play important roles in IgE-dependent MC activation [16].

**Figure 1:**
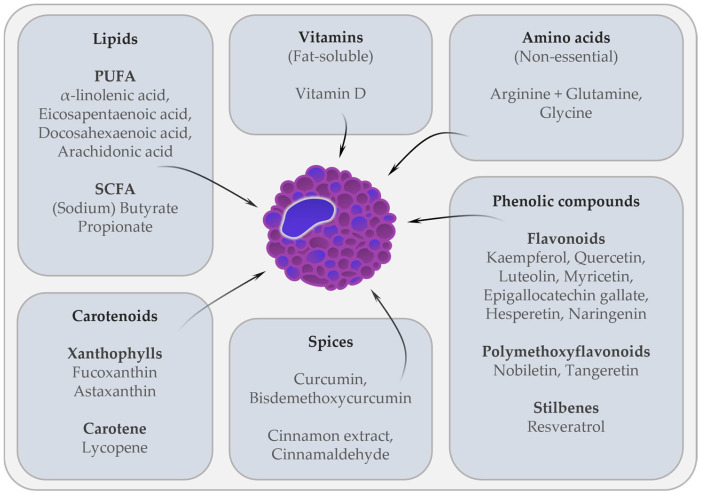
Dietary compounds with immunomodulatory effects on mast cells (MC). These dietary components were found to have anti-inflammatory effects on MC-mediated allergic reactions in vitro, and partly in vivo or in randomized controlled trials (RCT). Abbreviations: PUFA, polyunsaturated fatty acid; SCFA, short chain fatty acid.

MC play a key role in the pathogenesis of allergic diseases, including allergic rhinitis, asthma, atopic dermatitis, food allergy, drug allergy, and the life-threatening systemic MC-mediated reaction known as anaphylaxis [10,17]. For the treatment or prevention of allergic diseases, and as an alternative to conventional therapy, so-called “nutraceuticals” could be of interest. The term “nutraceutical” was coined by DeFelice in 1989 from the words “nutrition” and “pharmaceutical” and is defined as “a food or part of a food that provides medical or health benefits, including the prevention and treatment of a disease”. According to this definition, nutraceuticals can be isolated nutrients, dietary supplements, herbal products, as well as processed products such as soups and beverages [18]. It is believed that in addition to their nutritional value, they may also have pharmaceutical benefits [19]. The food sources used as nutraceuticals can be classified as dietary fiber, prebiotics/probiotics, polyunsaturated fatty acids, antioxidant vitamins, polyphenols, and spices [20]. Various naturally-occurring secondary plant compounds and other dietary components are known for their effect on MC activation. These include dietary substances such as certain fatty acids and amino acids, vitamins, antioxidant plant substances including carotenoids and flavonoids, but also various types of spices [21]. Figure 1 shows dietary components with potential immunomodulatory effects on MC-mediated allergic reactions.

The effects of these dietary components on MC activation can be studied in vitro in rodent and human MC models and in vivo on rodent animal models of allergic diseases (Table 1 and Table 2). There are several MC lines. However, they all have limitations. The rat basophilic leukemia 2H3 (RBL-2H3) cell line is widely used as MC line but shows characteristics of both MC and basophils and is not a true representation of either cell type. The murine mast cell line 9 (MC/9) serves as a model for IL-3-dependent mucosal MC in mice but has limited characteristic features of MC [22]. The human mast cell line-1 (HMC-1) consists of two subcolones; HMC-1.1 and HMC-1.2, which do not express functionally activatable FcεRI receptors [23,24]. Human Laboratory of Allergic Disease 2 (LAD2) cells express functionally activatable FcεRI receptors but tend to lose this property over time [25]. In addition to cell lines, murine bone marrow-derived mast cells (BMMC) are often used. They are derived from precursor cells in the bone marrow cells by culturing with IL-3 and/or stem cell factor (SCF) [26]. Similarly, human cord blood-derived mast cells (CBMC) and human peripheral blood mononuclear cell-derived mast cells (PBMCMC) originate from progenitor cells isolated from human umbilical cord blood or human buffy coat from peripheral blood by culturing with growth factors such as SCF [27]. Lastly, primary human skin mast cells (hsMC) and human intestinal mast cells (hiMC) are mechanically and enzymatically isolated from human skin or intestinal mucosa and purified by magnetic cell separation of c-Kit+ (CD117) cells. C-Kit is the receptor for the MC growth factor SCF [28,29]. Various rodent laboratory strains, including BALB/c, C57BL/6J, NC/Nga, ICR mice, and Sprague-Dawley rats, are used for the study of allergic diseases through specific treatments [30,31,32]. The generation of distinct allergy models by specific treatment and stimulation of animal models is listed in Table 2.

Because of the potential effects of these dietary components on MC in vitro and in vivo (Figure 1), they could potentially be used as nutraceuticals, providing an alternative or additional form of therapy for allergic diseases.

## 2. Effects of Dietary Components on MC In Vitro and In Vivo

Table 1 and Table 2 summarize the dietary components analyzed, their concentrations, the MC or animal model studied, and their effects on signaling molecules, preformed, and de novo synthesized mediators of MC.

### 2.1. Fatty Acids

Fatty acids are crucial components of lipid membranes and can influence the production and secretion of mediators by MC [94]. In particular, the long-chain n-3 polyunsaturated fatty acids alpha-linolenic acid (ALA), eicosapentaenoic acid (EPA) and docosahexaenoic acid (DHA), naturally found in fatty fish such as salmon or herring [95], exhibit anti-inflammatory properties [96]. Using LAD2 cells, Ding et al. (2021) reported a dose-dependent attenuation of C48/80-induced β-hexosaminidase (β-hex) and histamine release. Here, the application of ALA resulted in a decrease of histamine degranulation by about 60%. ALA also attenuated the C48/80-induced release of the cytokines C-X-C motif chemokine ligand (CXCL)-8/IL-8, IL-13, and tumor necrosis factor α (TNF-α) by LAD2 cells. These reductions in MC activity were associated with the inhibition of Lck/Yes-related novel protein tyrosine kinase (Lyn) activity in LAD2 cells via the Lyn-phospholipase Cγ (PLCγ)-inositol trisphosphate receptor (IP3R)-Ca^2+^ and Lyn-p38/NF-κB signaling pathways. Similarly, ALA exerted the same effects on serum histamine and cytokine release while reducing vasodilation and the percentage of degranulated MC in C57BL/6 mice [33].

EPA and DHA, which can be synthesized in the body from ALA, reduced the release of β-hex (≈25%) and cysteinyl-leukotriene (cys-LT) (≈80%) by LAD2 cells [34]. EPA and DHA thereby reduced the localization of the high affinity IgE receptor FcεRI in lipid rafts and decreased the phosphorylation of Lyn, spleen tyrosine kinase (Syk), and linker for activation of T cells (LAT) signaling molecules in IgE/antigen-stimulated LAD2 cells. G-protein-coupled-receptor (GPR)120, also known as free fatty acid receptor 4, was found to be expressed at both mRNA and protein levels in LAD2 cells, suggesting its involvement in mediating the inhibitory effects of PUFA on MC activation.

EPA and DHA may bind to and activate GPR120 in MC, potentially leading to increased cyclic adenosine monophosphate levels and the activation of protein kinase A, which could suppress MC degranulation [34]. Furthermore, ALA, EPA, and DHA inhibited the production of Th2-associated cytokines IL-4, IL-5, and IL-13 in activated BMMC and MC/9 by suppressing the nuclear expression of the transcription factors GATA binding protein (GATA) 1 and 2 [35]. GATA-1 and GATA-2 are involved in the activation of Th2-associated cytokine gene expression in MC [97,98]. P815 MC lacking GATA-1 showed limited or no effect on Th2-associated cytokine production following phorbol-12-myristate-13-acetate (PMA) and ionomycin (IONO) stimulation, unlike MC/9 or BMMC [35]. These findings suggest that GATA-1 is required for the downregulation of Th2 cytokine expression by omega-3 fatty acids, particularly for the Th2 cytokine IL-5. Oral administration of fish oil rich in omega-3 fatty acids reduced symptoms such as thickening of the epidermis and dermis and infiltration of MC and eosinophils in an atopic dermatitis mouse model [35]. In HMC-1, EPA and DHA reduced the secretion of the inflammatory mediators prostaglandin (PG)D_2_, IL-4, and IL-13 by more than 60%. The inhibition of IL-4 and IL-13 secretion was hereby correlated with a reduced generation of reactive oxygen species and suppression of MAPK. On the other hand, the omega-6 fatty acid arachidonic acid (AA) enhanced the production of proinflammatory MC mediators, including increased secretion of PGD_2_ and TNF-α by HMC-1. These findings suggest that EPA and DHA as omega-3 fatty acids may have beneficial effects in modulating MC function and reducing inflammation, whereas omega-6 fatty acids such as arachidonic acid may have pro-inflammatory effects [36].

MC in the gut and vascularized tissues are exposed to high concentrations of SCFA [77]. SCFA butyrate suppressed IgE-mediated BMMC release of TNF-α and IL-6 (≈70%) in a FcεRI-dependent manner. Phosphorylation of MAPK p38, extracellular-signal regulated kinase (ERK 1/2), and c-Jun N-terminal kinase (JNK) was downregulated by butyrate. In addition, butyrate treatment increased acetylation in the promoters of TNF-α and IL-6, but it blocked the binding of RNA polymerase II to these genes, resulting in suppressed transcription initiation. This suggests that butyrate modulates gene expression rather than receptor stimulation [37]. Furthermore, both SCFA propionate and butyrate, but not acetate, inhibited β-hex degranulation by about 90% in IgE/antigen-induced BMMC and PBMCMC. This inhibitory effect was independent of GPR41, GPR43, and peroxisome proliferator-activated receptors. Instead, butyrate affected histone deacetylase (HDAC) activity, similar to the HDAC inhibitor trichostatin A, leading to transcriptional silencing of the important FcεRI signaling genes bruton’s tyrosine kinase (Btk), Syk, and LAT [38].

Oral supplementation of sodium butyrate to weaned piglets reduced histamine release in the jejunal mucosa by about 40%. Gene expression and release of proinflammatory cytokines, including IL-6, TNF-α, and IL-13, decreased. The phosphorylation ratio of the MAPK JNK showed a decrease of about 60% after sodium butyrate feeding. However, no significant changes in the phosphorylation of ERK and p38 were observed. These results suggest that sodium butyrate improves the intestinal barrier function in weaned pigs by inhibiting the JNK signaling pathway [37]. This supports the findings of Folkerts et al. (2020) [38], as JNK is directly downstream of Btk, Syk, and LAT [77].

### 2.2. Amino Acids

The amino acids glutamine, arginine, and glycine have immunomodulatory and anti-inflammatory properties [39,40]. We showed that combined challenge of hiMC with pharmacological doses of the non-essential amino acids glutamine and arginine reduced leukotriene C_4_ (LTC_4_) secretion by about 45%, but not β-hex release. High-dose treatment with both amino acids decreased chemokine expression of CC chemokine ligand (CCL)-2, CCL4, CXCL8, and TNF-α up to 50% in hiMC. Arginine and glutamine acted by suppressing the phosphorylation of the MAPK ERK, p38, and JNK, as well as of protein kinase B (Akt). Since the release and expression of the aforementioned MC mediators is regulated in part by the activation of MAPK and Akt, arginine and glutamine may modulate the inflammatory responses of MC [39]. Importantly, the amino acid L-glutamine, but not D-glutamine, promoted MC activation in the intestinal mucosal during fat absorption, resulting in increased levels of rat mucosal mast cell protease II (RMCPII), histamine (≈60%) and PGD_2_ (≈70%) in response to dietary fat. This suggests that L-glutamine, but not D-glutamine, may accelerate proinflammatory responses leading to mucosal MC activation [78]. 

The amino acid glycine reduced the secretion of the proinflammatory cytokines TNF-α, IL-4, and IL-13, as detected in RBL-2H3 cells after IgE-mediated stimulation. Notably, no cellular degranulation was observed [40]. Oral administration of the amino acid glycine reduced mouse mast cell protease-1 (mMCP-1) and whey-specific IgE1 antibody serum levels in the serum and jejunum of a mouse model of cow’s milk whey allergy. Adequate glycine intake may be a mediator against (whey-induced) hypersensitivity reactions [40].

### 2.3. Vitamin D

Vitamin D is a fat-soluble vitamin with physiological and immunomodulatory properties [41,99]. It is found in animal foods such as fatty fish, egg yolk, liver, milk, and butter [100]. The main source of Vitamin D is the skin through sunlight [101]. BMMC, HMC-1, RBL-2H3, and P815 cultured without calcitriol-released histamine and TNF-α, while calcitriol inhibited their release by about 90–100%, suggesting that MC are automatically activated in a vitamin D deficient environment. Calcitriol also increased the expression of the vitamin D receptor (VDR) in MC. VDR bound to Lyn and blocked its interaction with FcεRI and myeloid differentiation primary response gene 88 (MyD88), attenuating downstream phosphorylation of Syk, activation of p38, and NF-κB in BMMC. In the presence of calcitriol, VDR also bound to the TNF-α promoter, thereby almost completely reducing TNF-α expression [41]. Yip et al. (2014) found that calcidiol (25(OH)D_3_) reduced IgE-mediated histamine release, cys-LT, and TNF-α by about 30% in BMMC in a VDR-dependent manner. The treatment of CBMC or PBMCMC with calcitriol or calcidiol resulted in a modestly reduced or comparable effect on the secretion of histamine, cys-LT, and TNF-α as in BMMC. MC 25-hydroxyvitamin D-1α-hydroxylase (CYP27B1) converted the intermediate metabolite calcidiol into calcitriol, which further suppressed MC activation. Calcidiol, although inactive, could bind to VDR with low affinity, but it is unclear whether this direct binding has suppressive effects on MC activation [27]. Pretreatment of LAD2 cells with calcidiol inhibited serum-induced vascular endothelial growth factor (VEGF) expression by suppressing the phosphatidylinositol-4,5-bisphosphate 3-kinase (PI3K)/Akt/p38 and MAPK/hypoxia-inducible factor 1-alpha (HIF-1α) signaling pathways [42]. HIF-1α regulates gene expression, including VEGF, which is produced by MC and eosinophil granulocytes and serves as an inflammatory cytokine [102]. VEGF is also a potential blood marker for chronic spontaneous urticaria (CSU). These findings suggest that adequate vitamin D supplementation may inhibit MC-dependent VEGF production and thus potentially alleviate the symptoms of CSU [42]. The application of the vitamin D metabolites calcitriol and calcidiol to the skin of mice reduced the extent of skin swelling in IgE-mediated passive cutaneous anaphylaxis (PCA) reactions. This response was dependent on MC-VDR and MC-CYP27B1 hydroxylation of calcidiol to biologically active calcitriol [27].

### 2.4. Carotenoids

Carotenoids are a broad group of natural pigments with a tetraterpene structure that possess anti-oxidant and anti-inflammatory properties [103]. Rich sources of carotenoids include dark green leafy vegetables, tomatoes, carrots, seaweed, and shellfish [104,105,106,107]. The effect of fifteen natural carotenoids on MC degranulation was investigated by administering them to RBL-2H3 prior IgE-mediated stimulation. Nine carotenoids, namely fucoxanthin, zeaxanthin, β-carotene, 3-hydroxyechinenone, astaxanthin, fucoxanthinol, lycopene, β-cryptoxanthin, and siphonaxanthin, reduced the IgE-mediated β-hex degranulation by about 70–30% [43]. Violaxanthin, phoenicoxanthin, canthaxanthin, neoxanthin, alloxanthin, and lutein did not show significant inhibitory effects. The ability of carotenoids to inhibit MC degranulation did not correlate with their chemical structures or cellular uptake. However, the underlying mechanism may involve the inhibition of antigen-induced translocation of IgE/FcεRI complexes into lipid rafts [43].

Oral fucoxanthin reduced mouse ear swelling induced by arachidonic acid, PMA, or oxazolone (OXA) by 20–28%, while percutaneous administration reduced it by more than 50% [79]. Both percutaneous fucoxanthin and oral fucoxanthin, which is metabolized to fucoxanthiol in the intestine to fucoxanthiol [108], effectively suppressed inflammatory responses either locally or systemically [79]. This suppressive effect of fucoxanthin and fucoxanthiol is attributed to their ability to suppress the mRNA expression of phospholipase A_2_ (PLA_2_), cyclooxygenase-2 (COX-2), and hyaluronidase in vitro [79]. 

Astaxanthin administration in PMA and calcium ionophore A23187 stimulated RBL-2H3 cells reduced the release of histamine and β-hex by about 30% [44]. In a murine model of contact dermatitis, topical application of astaxanthin reduced ear thickness and weight along with a decrease in TNF-α and IFN-γ levels in the ear skin [44]. Topical application of free astaxanthin in a phthalic anhydride (PA)-induced atopic dermatitis mouse model reduced the MC infiltration into the dermis. MC numbers (≈70%), serum TNF-α, IL-6, IL-1β cytokines, and IgE concentrations were also reduced [80,81]. In addition, astaxanthin suppressed inducible nitric oxide synthase (iNOS), COX-2 expression [81], and malondialdehyde production [46], indicating reduced oxidative stress in atopic dermatitis mice. Conversely, astaxanthin treatment increased the levels of antioxidant biomarkers such as glutathione (GSH), glutathione peroxidase (GPx-1), and heme oxygenase 1 (HO-1) [80,81]. In contrast, oral administration of astaxanthin reduced the total number of MC (≈50%) less effectively than topical administration (≈70%) [80] and did not alter the expression levels of TNF-α and IL-1β [83]. A study by Lee et al. (2020) demonstrated that topical liposomal astaxanthin treatment showed greater efficacy in preventing inflammatory cytokine release and oxidative stress in a PA-induced atopic dermatitis model by about 20–30% compared to free astaxanthin [82]. In terms of signaling pathways, astaxanthin treatment inhibited the activation of STAT3 and NF-κB [81,82].

The carotene lycopene reduced the cytokine expression of IL-4, IL-13, and IL-9 by about 60% in an ovalbumin (OVA)-induced food allergy model [84]. IL-9 has been shown to increase the number of intestinal MC in food allergy [109]. However, lycopene supplementation reduced the number of MC in the colonic lamina propria by about 50% [84].

### 2.5. Flavonoids

Flavonoids are a group of naturally-occurring polyphenolic plant substances with antioxidant and anti-inflammatory properties [110,111,112]. The flavonols kaempferol, quercetin, luteolin, and myricetin inhibited IgE-, C48/80-, or streptavidin-mediated MC degranulation and cytokine release in LAD2 cells [45,46,49,50,52,54]. Quercetin, present in onions, oregano, and berries [113], had the strongest inhibitory effect on histamine (≈80%), IL-8, and monocyte chemoattractant protein-1 (MCP-1/CCL2) release in C48/80 or IgE/antigen-stimulated LAD2 cells [49,50]. Luteolin, at a dose of 20 µM, had a greater inhibitory effect on histamine release (≈60%) than kaempferol (≈50%) at 100 µM [46,54]. Luteolin also inhibited PGD_2_ release by about 40% in LAD2 cells [54]. Kaempferol and quercetin inhibited IgE-mediated LAD2 cells activation, likely through the inhibition of Lyn activity and PLCγ-IP3R/Ca^2+^ downstream signaling [45,46,49,50]. Cao et al. (2020) even showed that kaempferol significantly prevented the translocation of Parkinson’s disease protein 7 (DJ-1) to the plasma membrane, thereby inhibiting the activation of Lyn and eventually restraining its receptor-distal signaling molecules involving Syk, Btk, PLCγ, IP3R, protein kinase C (PKC), MAPK, Akt, and NF-κB in LAD2 cells [46]. Luteolin, however, had no inhibitory effect on Lyn but decreased PLCγ phosphorylation and intracellular calcium concentration mediated by FcεRI and MRGPRX2 [54]. Quercetin was found to have good binding affinity to MRGPRX2, similar to the one of C48/80 or substance P, and attenuated MRGPRX2-mediated pseudo-allergic responses [50].

In RBL-2H3 cells, Aceriphyllum rossii (ARE), a substance rich in quercetin and kaempferol, inhibited the histamine release by about 40% and PGE_2_ release almost completely [51]. ARE also reduced the release of the cytokines TNF-α (≈40%) and IL-4 (≈20%) [51], but reduced this less than myricetin (≈60%) alone [53]. Kaempferol alone reduced histamine release by about 70% and almost completely inhibited PGF2α release in stimulated RBL-2H3 cells [47]. A higher dose of kaempferol of 50 µM in IL-33-stimulated BMMC inhibited cytokine release of TNF-α, IL-6, and IL-13 by almost 80% [48]. IgE/antigen-stimulated activation of RBL-2H3 cells was inhibited by kaempferol, quercetin, ARE, and myricetin via the Syk pathway [51,53]. IL-33-stimulated BMMC reduced PLCγ and cell surface expression of FcεRI time-dependently but increased the expression of Src homology 2 domain-containing inositol 5-phosphatase 1 (SHIP1) in response to kaempferol treatment. SHIP1 was also upregulated in mouse peritoneal MC, which may have anti-allergic effects [48]. Luteolin, naturally occurring in celery, parsley, and broccoli [114], reduced the release of TNF-α and IL-1β in HMC-1 by almost 80% [55]. IL-33-stimulated HMC-1.2 showed a reduction of IL-31 by about 70% in response to luteolin due to a possible inhibition of the IL-31/IL-33 axis. This could alleviate IL-33-activated diseases such as asthma [56]. 

Epigallocatechin gallate (EGCG), a tea catechin, reduced both histamine degranulation and cytokine release (TSLP, IL-1β, IL-6, IL-8) in receptor activator of NF-κB ligand (RANKL)-stimulated HMC-1 cells by targeting PI3K, MAPK, caspase-1, and NF-κB signaling cascades [57]. Oral EGCG reduced sneezing episodes, nasal rubbing, serum histamine levels (≈30%), and nasal mucosal expression of inflammatory molecules COX-2, IL-1β, IL-4, and IL-6 in a mouse model of allergic rhinitis [85]. 

(-)-Epigallocatechin-3-*O*-(3-*O*-methyl) gallate (EGCG″Me), an *O*-methylated catechin and found in the Benifuuki green tea, showed no significant effect on MC degranulation in IgE/antigen-activated RBL-2H3 cells but did when the bioactive flavanone eriodictyol or the citrus flavanone hesperetin was added. Combined administration of EGCG3″Me with eriodictyol or hesperetin inhibited IgE-mediated degranulation by inducing 67-kDa laminin receptor/soluble guanylate cyclase acid/acid sphingomyelinase signaling and enhancing this pathway. In a PCA mouse model both eriodictyol and highly absorbable α-glucosyl hesperidin potentiated the anti-allergic effect of Benifuuki green tea, with eriodictyol having a stronger effect on PCA responses (≈50%) [58,59].

The flavone naringenin, found in citrus fruits, is characterized by its antioxidant properties and immunomodulatory effects [115,116]. Naringenin-attenuated TSLP-promoted MC proliferation by downregulating phosphorylated signal transducer and activator of transcription 6 (pSTAT6) and murine double minute 2 (MDM2) and upregulating poly ADP-ribose polymerase (PARP) cleavage and p53 activation in HMC-1. MC-mediated inflammatory responses were suppressed by naringenin by downregulating TSLP-induced increase in IL-13 and TNF-α levels by almost 40% in MC. Therefore, naringenin may have an antiproliferative effect by regulating the levels of anti-apoptotic and proapoptotic factors in MC [60]. In an OVA-induced allergic rhinitis model, oral treatment with naringenin improved nasal symptoms and decreased serum total IgE and Th2 cytokines IL-4 and IL-5 compared with the OVA treated group [86]. Intraperitoneal administration of naringenin reduced the development of ear swelling and skin lesions in a murine model of atopic dermatitis. These effects may be the result of reduced lesion infiltration by CD4^+^ T cells, CD8^+^ T cells, degranulated MC (≈40%), suppression of serum IgE levels, and a reduced production of IFN-γ by activated CD4^+^ T cells [87].

Kaempferol, abundant in spinach and kale [113], reduced OVA [46] and C48/80 [45]-induced symptoms of PCA in C57BL/6 mice by suppressing serum histamine (≈80%), TNF-α, IL-8, and MCP-1 levels. Here, the process of regulation of PLCγ phosphorylation leading to calcium mobilization by kaempferol contributed to the suppressive effects on pseudo-allergic reactions [45,46]. Quercetin attenuated hind paw thickness and serum histamine (≈40%) release in an C48/80-induced pseudo allergy mouse model. Additionally, quercetin reduced serum MCP-1 and IL-8 levels in mice. Due to a reduced percentage of degranulated MC and histamine, histamine-induced vasodilation was inhibited, indicating the anti-pseudo allergic effects of quercetin [50]. In an OVA-induced model of allergic conjunctivitis, quercetin inhibited the expression of serum histamine (≈60%), IL-4 (≈90%), and TNF-α in the peripheral blood. Furthermore, the OVA-induced number of degranulated MC (≈50%), eosinophil levels, and vascular permeability were attenuated. Quercetin may have acted through its ability to inhibit Lyn/ERK1/2, PLCγ/IP3R-Ca^2+^, and Lyn/NF-κB signaling [49].

### 2.6. Polymethoxyflavonoids

Citrus peel polymethoxyflavonoids, such as nobiletin and tangeretin [117], are a group of citrus flavonoids that have anti-cancer and anti-inflammatory properties [118,119]. We have shown that both nobiletin and tangeretin inhibited the expression of CXCL8, CCL2, CCL3, CCL4, and IL-1β upon LPS-mediated stimulation in hiMC. CXCL8, CCL2, CCL3, CCL4, and TNF expression decreased after nobiletin and tangeretin treatment of IgE-mediated stimulated hiMC. Also, tangeretin reduced IgE-mediated IL-1β expression. IgE-mediated MC degranulation of β-hex and LTC_4_ was reduced in response to nobiletin, but not in response to tangeretin. Both nobiletin and tangeretin suppressed ERK1/2 phosphorylation upon IgE-mediated stimulation. The potentially affected pathway involved ERK in IgE-dependent stimulation and NF-κB in LPS-mediated activation [61]. In PMA or histamine-stimulated RBL-2H3 cells, nobiletin and tangeretin inhibited the IL-4 and TNF-α expression by about 60% and 50%, respectively. The activation of the transcription factors NF-κB, c-Jun, and p38 was suppressed after histamine-induced activation of RBL-2H3 cells. Furthermore, PKC activity was inhibited [62]. Nobiletin and tangeretin inhibited histamine- and C48/80-induced scratching behavior and histamine-induced vascular permeability in ICR mice by about 50%. In addition, histamine-induced expression of IL-4 and TNF-α in the mouse skin was almost completely inhibited. In terms of signaling, the transcription factor NF-κB, which regulates TNF-α expression, and AP-1, which regulates IL-4 expression, were suppressed. These results suggest that nobiletin and tangeretin may inhibit MC secretion by suppressing the activation of transcription factors activator protein-1 (AP-1) and NF-κB [62].

### 2.7. Resveratrol

Resveratrol belongs to the group of polyphenols, precisely the stilbenes [120], and is best known for its antioxidant [121] and anti-inflammatory [122] effects in vitro and in vivo. Resveratrol is found in various fruits such as berries, and is especially present in the skin of grapes [123,124,125]. We could show that pretreatment with 50 µM resveratrol reduced the IgE-mediated β-hex release in hiMC by about 50% and led to a complete inhibition after treatment with 100 µM resveratrol. The mRNA expression of the chemokines CXCL8, CCL2, CCL3, CCL4, and TNF-α decreased in a dose-dependent manner until complete inhibition in response to 100 μM. Resveratrol thereby suppressed IgE-mediated phosphorylation of both nuclear and mitochondrial STAT3 and ERK1/2 by almost 100%. Thus, we concluded that resveratrol prevents MC activation and cytokine expression by inhibiting this pathway [16]. Resveratrol (100 μM) treatment in IgE/antigen-stimulated hsMC decreased the secretion of β-hex by about 80%, as well as the secretion of the de novo synthesized mediators PGD_2_, TNF, and IL-6. Interestingly, low concentrations of resveratrol (<10 µM) enhanced TNF production in hsMC after FcεRI cross-linking [63]. In contrast, using a lower concentration of resveratrol (10 µM) in BMMC, the IgE-mediated release of TNF-α and IL-6 was already reduced by about 70% and the release of the eicosanoids LTC_4_ and PGD_2_ by more than 80%. The BMMC degranulation of β-hex was attenuated by about 60%. Resveratrol decreased the phosphorylation of protein tyrosine phosphatase 1B (PTB1B) and Syk, thereby inhibiting FcεRI-dependent MC activation by regulating the Syk pathway [65].

In either IL-33- or IgE/antigen-stimulated BMMC, treatment with 25 µM resveratrol reduced the release of TNF-α, IL-13, and IL-6 by more than 40%. The underlying mechanism may be the regulation of the MAPK-activated protein kinase (MK)-2/3-PI3K/Akt axis, as IL-33-induced IL-6 and IL-13 production in MC is mediated by MK2/3 mediated activation of the PI3K/Akt pathway [64]. 

RBL-2H3 cells stimulated with either IL-33 [66] or IgE/antigen [67] and treated with resveratrol suppressed the release of proinflammatory cytokines and chemokines, such as TNF-α, IL-6, IL-4, IL-3, and MCP-1. This effect could be explained by the reduced phosphorylation of p38, ERK, and JNK after resveratrol treatment [67]. In addition to MAPK, the incubation of IL-33- and IgE/antigen-stimulated RBL-2H3 cells with resveratrol reduced phosphorylation of p38, inhibitor of nuclear factor kappa B (IκBα), and NF-κB subunit p65 by more than 50% [66,67]. 

A higher dose of resveratrol (200 µM) showed a dose-dependent attenuation of C48/80-induced β-hex and histamine release by about 80% in the LAD2 MC line. Cytokine expression of MCP-1, TNF-α, and IL-1β was inhibited by resveratrol by about 40%, 60%, and 80%, respectively. Resveratrol administration increased the expression of nuclear erythroid 2-related factor 2 (Nrf2) and the generation of its target genes transcription factor HO-1 and NADPH dehydrogenase quinone 1 (NQO1). Thus, the Nrf2/HO-1 pathway may serve as a target for the therapy of MC-mediated allergic disorders [68]. Using HMC-1, Moon et al. (2020) [69] demonstrated a reduction of PMA and calcium ionophore A23187-mediated TSLP mRNA expression after pretreatment with resveratrol. Furthermore, decreased intracellular calcium levels resulted in a reduced production of receptor interacting protein (RIP) 2/caspase-1, which suppressed the phosphorylation of IκBα and activation of NF-κB by about 50%. This mechanism may be responsible for the suppression of TSLP production by resveratrol, which plays an important role in the pathogenesis of atopic diseases such as allergic rhinitis [69].

In an OVA-induced food allergy mouse model, resveratrol reduced the serum histamine and IgE levels, as well as MCP-1, by about 50% [88]. We found that resveratrol treatment prevented the increase in mast cells in OVA-induced allergic enteritis in both the duodenum and colon and delayed the onset of disease symptoms [89]. In a murine PCA model, the administration of resveratrol reduced plasma histamine levels by about 50% and attenuated tissue activation of Syk, PLCγ, and PKCµ [126]. Furthermore, resveratrol reduced plasma levels of IL-6, IL-13, MCP-1, and TNF-α by about 50% in IL-33-stimulated Sprague-Dawley rats [66]. Consistent with this, serum levels of TNF-α, IL-1β, IL-18, and intestinal β-hex levels were reduced by about 50% in Sprague-Dawley rats suffering from ischemia-reperfusion (IIR) after treatment with resveratrol [90]. In a C48/80-induced model of pseudo allergy, resveratrol dose-dependently decreased serum levels of histamine and MCP-1, TNF-α, and IL-8. In addition, the number of degranulated MC was reduced by about 70% at the highest application concentration of resveratrol of 20 mg/kg [68]. The application of resveratrol to OVA-treated mouse skin normalized OVA-mediated epidermal thickening and reduced MC activation and chemokine (CCL2 and CCL5) expression in skin tissue. The mechanism of action involved inhibition of sphingosine kinase 1 (SphK1), a sphingosine-1-phosphate (S1P)-producing enzyme, along with STAT3 and NF-κBp65, which are involved in chemokine production [91]. S1P drives MC activation, which triggers STAT3 activation and the subsequent release of the cell-recruiting chemokines CCL2, CCL3, and CCL5 [127,128].

### 2.8. Spices

#### 2.8.1. Curcumin

Curcumin and bisdemethoxycurcumin (BDMC) are two natural compounds found in curcuma, also known as turmeric. Both compounds have been shown to have anti-inflammatory properties [93,129,130]. The identification of other anti-inflammatory components of turmeric is part of current research [131,132]. In IgE/antigen-stimulated RBL-2H3 cells, curcumin showed a reduction in β-hex (≈80%) and histamine release (≈60%) [70], while BDMC showed a similar reduction in β-hex release at the same concentration [93]. Using HMC-1, Zhang et al. (2015) [72] and Kong et al. (2018) [73] were able to detect a reduction in the inflammatory cytokines TNF-α, IL-6, and IL-8, by more than 60% after both curcumin and BDMC treatment. Curcumin treatment of IgE/antigen-stimulated BMMC showed a reduction of LTC_4_ and PGD_2_ expression of about 80% [71]. Curcumin and BDMC may act by inhibiting the downstream Syk pathway, thereby suppressing the activation of MAPK ERK, JNK, and p38, as well as NF-κB and translocation of PKC-δ [70,71,72,73,93]. Recently, the antipruritic effect of curcumin was found to be mediated through the Mas-related G-protein-coupled receptor B2 (MRGPRB2), the rodent homolog of the human MRGPRX2, as demonstrated by inhibition of C48/80-induced calcium influx in mouse peritoneal MC in a concentration-dependent manner. In addition, molecular docking results showed that curcumin has affinity for the ligand pocket of the MRGPRX2 protein, indicating that curcumin can inhibit the activation of MRGPRX2 [133]. These findings support the involvement of the MRGPRB2/X2 receptor in the mechanism of action of curcumin action against pruritus.

The oral administration of curcumin reduced serum histamine, PGD_2_, and LTC_4_ (≈50%) levels in a mouse model of passive systematic anaphylaxis [71]. Curcumin showed a greater effect on histamine (≈70%) and OVA-specific IgE (≈80%) reduction compared to BDMC, demonstrating a less than 50% reduction in both parameters in OVA-stimulated BALB/c mice [72,92,93]. Curcumin treatment in a mouse model of allergic rhinitis reduced TNF-α cytokine levels by about 70% by suppressing the phosphorylation of the Src kinases Fyn, Lyn, and Syk [72]. Conversely, treatment with BDMC in a mouse model of food allergy reduced serum levels of the Th2 cytokines IL-4, IL-5, and IL-13 by less than 30%, while increasing IFN-γ levels. Consequently, the levels of GATA-3 protein, a critical transcription factor involved in Th2 immune responses, were downregulated. These effects were accompanied by inhibition of the MAPK signaling pathway and the nuclear translocation of NF-κB. This mechanism may be responsible for the reduced anaphylaxis symptoms, lower diarrhea scores, and attenuated increase in rectal temperature in mice after OVA stimulation [92].

#### 2.8.2. Cinnamon Extract

Cinnamon extract (CE) and its compounds can be obtained from cinnamon bark and can have anti-inflammatory effects on MC [74,75]. CE treatment on IgE/antigen-stimulated RBL-2H3 and hiMC resulted in a downregulation of β-hex and cys-LT degranulation by about 80%. The release of de novo synthesized proinflammatory mediators CXCL8, CCL2, CCL3, CCL4, and TNF in hiMC was almost completely (≈90–100%) inhibited [74]. In vivo treatment of mice with oral CE also resulted in a downregulation of the expression of rodent MC proteases carboxypeptidase A (MC-CPA) and MC tryptase (MCP6) in the mouse duodenum. A similar downregulation of tryptase expression was observed in vitro in hiMC cells. Regarding the possible mechanism of action, the decreased phosphorylation of ERK, p38, and JNK, as well as of Akt, was observed [74]. 

In addition, we found that cinnamaldehyde (CA) is the main mediator of CE in MC inhibition [75]. RBL-2H3 cells treated with CA prior to IgE-dependent or IgE-independent stimulation attenuated β-hex degranulation by about 90%. In hiMC cells, CA significantly decreased the release of β-hex and almost completely inhibited the release of LTC_4_ and CXCL8. IgE-mediated expression of CXCL8, CCL2, CCL3, and CCL4 were downregulated in hiMC. Similar inhibitory effects of CE and CA were observed, except for the expression of the MC proteases tryptase and chymase, which remained unaffected after CA treatment. ERK phosphorylation was reduced in both RBL-2H3 and hiMC, and PLCγ1 phosphorylation was additionally downregulated in RBL-2H3, both of which are important signaling proteins of MC activation [75]. 4-chlorocinnamaldehyde and 4-trifluoro-cinnamaldehyde, derivatives of cinnamaldehyde, inhibited antigen-mediated β-hex release, and mRNA expression of IL-4 and TNF-α in RBL-2H3 cells [76]. 4-chlorocinnamaldehyde reduced the phosphorylation of p38, ERK, and JNK, as well as MEK-1/2 and MKK-4, following stimulation with PMA and calcium ionophore A23187 [76]. These MAPK are involved in the activation of transcription factors of inflammatory cytokines [134]. Therefore, both 4-chlorocinnamaldehyde and 4-trifluoro-cinnamaldehyde may regulate MC activity by inhibiting the MKK-MAPK signaling pathway [76].

**Figure 2:**
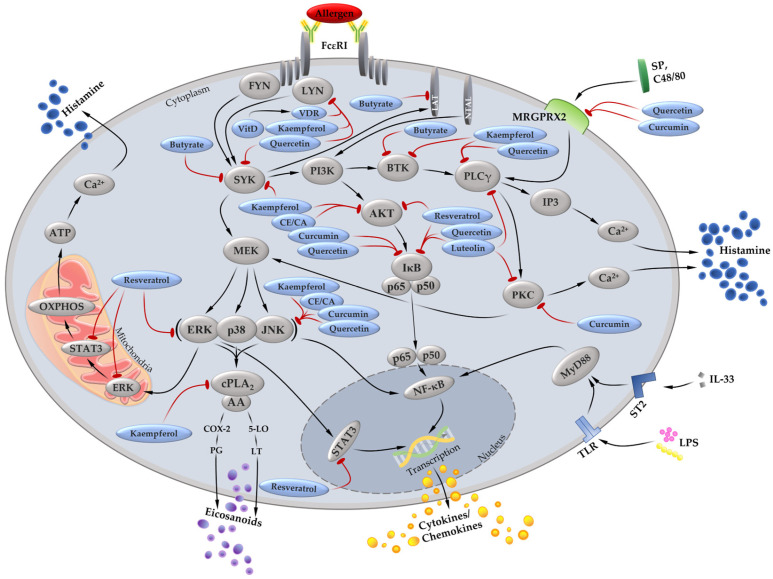
Signaling molecules in MC described to be affected by butyrate, vitamin D, the phenolic compounds kaempferol, quercetin, luteolin, and resveratrol, as well as the spices curcumin, cinnamon extract (CE), or cinnamaldehyde (CA). As stated in the text, these dietary components (blue ellipses) showed a potent suppressive effect on the release of histamine, cytokines/chemokines, or eicosanoids due to their influence on the signaling molecules (grey ellipses) of MC. The red arrows indicate the suppressive effects on signaling molecules.

Figure 2 shows signaling molecules in MC affected by butyrate, vitamin D, the phenolic compounds kaempferol, quercetin, luteolin, and resveratrol, as well as the spices curcumin, cinnamon extract, or cinnamaldehyde.

## 3. Effect of Dietary Components on Allergic Diseases in Randomized Controlled Trials

In addition to in vitro and in vivo studies, the effects of dietary components on allergic diseases have also been reported in randomized controlled trials (RCT) [135]. Table 3 summarizes the dietary components analyzed, their dosage, study type, duration, population, and their effect on MC-associated allergic diseases in children and adults in RCT.

Mild seasonal pollen allergic rhinitis (AR) treated with intranasal vitamin D adjuvant therapy showed a reduction in AR symptoms (≈40%), serum IL-4, and peripheral blood eosinophils compared to desloratadine citrate disodium (DCD) treatment alone. Serum calcidiol (25(OH)D_3_) levels were increased by about 50%. Overall, adjuvant vitamin D therapy increased the effective rate of DCD from 84% to 97% [136]. Furthermore, adult patients with AR and vitamin D deficiency treated with vitamin D and cetirizine showed reduced scores for rhinorrhea, nasal itching, sneezing, and postnasal drip. Again, the serum 25(OH)D_3_ levels were elevated by 60% after vitamin D supplementation. In addition, adjuvant vitamin D supplementation reduced symptom severity in patients with AR. Interestingly, all significant effects were observed after eight weeks of treatment compared to four weeks of treatment, indicating the time span for vitamin D to induce its immunologic effects [137]. Vitamin D may be an effective adjuvant in AR patients by suppressing inflammatory mediators, cells, and clinical symptoms.

Oral administration of the flavonoid quercetin to adult patients with allergic symptoms of AR resulted in a reduction in total symptom score (≈27%), sleeping disturbance (≈40%), and pruritus other than ocular/nasal. Further, the number of eosinophils in nasal discharge was reduced [138].

The daily consumption of Benifuuki green tea containing *O*-methylated EGCG reduced symptoms of Japanese cedar pollinosis. Oral consumption of 700 mL green tea reduced symptoms of runny nose, itchy eyes, tearing, total nasal and ocular symptom score, and nasal and ocular symptom-medication score by less than 20% during the pollen season. Moreover, the peripheral eosinophil counts were reduced after pollen exposure [139]. Although the results of this study are statistically significant, the overall effects of Benifuuki or the intake of *O*-methylated EGCG may not be strong enough to be an alternative to pharmacological treatment. However, the daily use of Benifuuki may reduce the symptoms of AR [139].

Adult AR patients treated with resveratrol showed a reduction in nasal symptoms compared to the placebo group. As a related effect, resveratrol reduced IgE, IL-4, TNF-α, and eosinophil levels in the blood of participants. Additionally, resveratrol treatment improved the quality of life of adults with AR [140]. Furthermore, intranasal administration of resveratrol in combination with carboxymethyl-β-glucan significantly attenuated nasal symptoms such as itching, sneezing, rhinorrhea, and congestion in children with pollen-induced AR [141]. 

The administration of curcumin in capsules to adults with perennial AR reduced nasal symptoms (≈60%) such as sneezing, itching, rhinorrhea, and nasal obstruction compared to the placebo group. In addition, curcumin decreased the cytokines levels of IL-4 and TNF-α in activated peripheral blood mononuclear cells (MNC) and IL-8 in activated polymorphonuclear neutrophils (PMN). Curcumin increased the production of IL-10 and soluble intercellular adhesion molecule-1 (sICAM-1), and improved the baseline nasal airflow in curcumin-treated patients with AR. However, curcumin had no effect on IFN-γ in MNC and PGE_2_ or LTC_4_ in PMN [142]. 

Adult patients with acute symptoms of AR were treated with cinnamon bark extract. After seven days, patients showed a decrease in nasal, eye, and non-nose/eye symptoms. In addition, the total white blood cell count and neutrophil count decreased after treatment with cinnamon bark extract [143]. In conclusion, both curcumin and cinnamon showed the ability to reduce AR symptoms by suppressing inflammatory mediators [142,143].

## 4. Discussion

As the prevalence of allergic diseases continues to increase, appropriate therapeutic and preventive strategies against them need to be developed [1]. In this context, MC play a crucial role, as they are the key effector cells of type 1 allergies [10]. Therefore, dietary components with immunomodulatory effects on MC could be of interest. These include secondary plant compounds such as carotenoids, flavonoids, polymethoxyflavonoids, and resveratrol, as well as spices. In addition, other components of the daily diet such as fatty acids, amino acids, and vitamins are found to have immunomodulatory effects on MC and thus on allergic diseases. Because of their effects, some of these food components could likely serve as nutraceuticals in the therapy of MC-associated allergic diseases.

The inhibitory effects of these nutrients on MC activation in vitro and in vivo are not always in the same range. Apart from their different effects on MC, different concentrations/triggers and MC/animal models were used depending on the experiment. This must be taken into account when comparing their effects on MC. In this review, we report that SCFA butyrate, vitamin D (calcitriol), polyphenols such as kaempferol, quercetin, luteolin, and resveratrol, as well as spices such as curcumin and cinnamon, exhibited the most potent inhibitory effects on MC degranulation, arachidonic acid metabolites, and cytokine release. These effects were observed in both in vitro and in vivo studies, highlighting the potential of these substances to modulate MC activity, and associated inflammatory responses. On average, the inhibitory effect of the dietary components on MC was lower in vivo than in vitro. In addition, these in vivo animal studies did not always clarify whether the dietary compounds inhibited MC directly or indirectly and what concentrations they reached in the MC. RCT were used to summarize the efficacy of the nutritional substances on MC-mediated allergic diseases.

RCT on the effects of vitamin D, quercetin, *O*-methylated EGCG, resveratrol, curcumin, and cinnamon on patients with AR could be included in this review. All of them showed an effect on the reduction of AR symptoms, inflammatory mediators, or immune cells. The oral administration of curcumin (≈60%) and nasal administration of cinnamon extract (≈50%) showed the strongest effects on the reduction of AR-specific symptoms [142,143]. Vitamin D, quercetin, *O*-methylated EGCG, and resveratrol reduced allergy symptoms by less than 40%, with *O*-methylated EGCG having the weakest effect (<20%) [136,137,138,139]. Nasal administration of resveratrol reduced approximately 80% of blood eosinophils in the blood, showing the strongest reduction of immune cells [140].

In both included studies, vitamin D was used as a supplement to the conventional medication. Thus, the combination of vitamin D and the antihistamine drug (97%) resulted in a more effective improvement in relative symptoms in AR patients compared with the antihistamine drug alone (84%). It can be concluded that vitamin D can be used as a nutraceutical in the adjuvant therapy of AR [136,137]. While oral administration of vitamin D showed effects after 2 months [137], nasal administration showed significant effects after one month [136]. This may be due to the lack of time for vitamin D to exert its immunological effects [137]. In comparison, nasal administration of cinnamon extract already showed anti-inflammatory effects after seven days [143]. However, the site of nutrient administration may also be a relevant factor contributing to faster effects [41]. The use of vitamin D nasal drops could bypass digestive tract enzymes and liver metabolism [136]. This could improve its bioavailability due to its low molecular weight (<4000 g/mol) [144]. In addition, the nasal mucosa has a high affinity for fat-soluble substances such as vitamin D [136]. Consequently, the site of administration may play an important role in the short-term relief of symptoms in allergic reactions.

Curcumin showed inhibitory effects on the secretion of PGD_2_ and LTC_4_ (≈50%) in in vitro and in vivo animal models [71], while no significant effect was found in the RCT [142]. To achieve an ≈50% inhibition of eicosanoids, mice were given 50 mg/kg curcumin per day orally [71], while, in the RCT, a concentration of 500 mg/day was used in patients with AR [142]. However, the oral administration of these nutrients through a normal diet may not be practical because the concentrations in foods or in turmeric root powder may not be sufficient. Patients may not achieve the required active doses at the MC because many dietary components have very low bioavailability—less than five percent [145].

In this context, “bioavailability” refers to the fraction of the ingested nutraceutical that is accessible for absorption at the level of the gastrointestinal tract (GIT), metabolized, and distributed to organs and tissues in the GIT [146]. Many substances are lost after oral administration, due to various factors. Endogenous and exogenous factors, such as the physicochemical properties of the nutraceuticals, the food matrix, food processing and storage can affect the bioavailability of nutraceuticals. Age, genetic characteristics, and health status of consumers are additional factors that influence food digestion and may also affect the oral bioavailability of nutraceuticals [145]. Solubility, lipophilicity, and permeability also affect the bioavailability of nutraceuticals. Lipophilic biomolecules such as vitamin D, carotenoids, or omega-3 PUFA are poorly soluble in the fluids of the digestive tract and have low bioavailability. In comparison, hydrophilic biomolecules such as catechins have high solubility but are more difficult to transport across the lipophilic bilayer of epithelial cells [147,148]. For example, EGCG has high solubility but poor cell membrane permeability, whereas curcumin has low solubility and low cell membrane permeability. Resveratrol has low solubility but high cell membrane permeability [145]. This highlights the importance of different delivery systems depending on the characteristics of the nutraceutical. Therefore, alternative delivery forms for nutraceuticals to increase their bioavailability have been analyzed to increase their bioavailability by improving these points and thus reducing the required doses.

One way to improve the poor bioavailability of nutraceuticals is to co-administer a potent bioenhancer, such as piperine, from black pepper [149]. Piperine is a known inhibitor of hepatic and intestinal glucuronidation and leads to an increase in the oral bioavailability of curcumin [150] and resveratrol [151]. However, the oral administration of curcumin-loaded nanoparticles showed increased oral bioavailability of curcumin in rats compared to curcumin with piperine [152].

The use of nanocarriers as delivery systems for nutraceuticals has been proven to be an effective method to increase the bioavailability of nutraceuticals [153]. Nanocarriers loaded with bioactive compounds have a very small size (1 to 100 nm), a very large interface, a surface electrical charge, and a variety of carrier materials. They improve the hydrosolubility of nutraceuticals, control their release in the GIT, prolong the residence time in the GIT, and improve intestinal permeation and transcellular delivery [154]. Nutraceuticals can be encapsulated using biopolymer nanocarriers as food-grade materials. These materials can be plant polysaccharides or food proteins [145]. For example, encapsulation in protein casein micelles protected vitamin D in gastric fluid and had four times higher bioavailability compared to free vitamin D [155]. Encapsulation of resveratrol in zein-based nanoparticles increased oral bioavailability by up to 50% and provided high and prolonged plasma levels of resveratrol over 48 h [156]. Another possibility is the packaging of nutraceuticals as lipid-based nanocarriers in nano-/or microemulsions, solid lipid nanoparticles (SLN), nanostructured lipid carriers (NLS), or polymeric micelles such as liposomes [145]. For example, the topical application of liposomal astaxanthin resulted in a greater reduction in proinflammatory mediators and atopic dermatitis-like skin inflammation than free astaxanthin in a mouse model [82]. Due to the poor water solubility of astaxanthin [157], the liposomal formulation improves water solubility by conjugation with phospholipid structures [158]. However, the efficacy of nano-based astaxanthin delivery systems has not yet been clinically tested [159]. Furthermore, the new lipid-based nanocarrier phytosome has been used to encapsulate quercetin for the treatment of AR [138]. This newly developed quercetin phytosome was able to achieve very high plasma levels of quercetin in volunteers—up to 20 times higher than the usual level of an orally administered dose of quercetin—and had no significant side effects [160]. Due to their small size and high surface area, nanoparticles allow nutraceuticals to penetrate deep into tissue and be efficiently absorbed by the cells. This results in a more efficient delivery of the substances to the physiological target site [161,162]. Therefore, research into improved delivery systems is critical for the use of nutraceuticals in allergic diseases.

## 5. Conclusions

The studies included in this overview suggest that dietary components such as fatty acids, amino acids, vitamins, carotenoids, flavonoids, and spices are able to attenuate proinflammatory, particularly IgE-dependent MC-mediated responses in vitro and in vivo. Overall, butyrate, vitamin D, the polyphenols kaempferol, quercetin, luteolin, and resveratrol, and the spices curcumin and cinnamon, were found to have the most potent anti-inflammatory effects on MC in vitro and in vivo. RCT reported beneficial effects of vitamin D, quercetin, *O*-methylated EGCG, resveratrol, curcumin, and cinnamon on allergic rhinitis symptoms and inflammatory cell reduction. In particular, the oral administration of curcumin and nasal administration of cinnamon extract have been identified as potential substances to reduce allergic symptoms of allergic rhinitis. However, the use of the dietary components as nutraceuticals in allergic diseases is limited by their poor bioavailability. There are several strategies, such as nanocarriers as delivery systems, to favorably influence the pharmacokinetics of these nutrients. Further clinical studies are needed to investigate their oral bioavailability and delivery systems. Nevertheless, these dietary components could be considered as nutraceuticals in future studies as potential adjuvants or alternative medicines for the prevention or treatment of allergic diseases.

## Figures and Tables

**Table 1:** Overview of the effects of dietary components on MC in vitro.

Substance	Dosage	MC Model	Stimulation	MC Degranulation	AA Metabolites	Cytokines/Chemokines	Signaling Molecules	Ref.
ALA	50, 100, 200 µM	LAD2	C48/80, SP	↓ β-hex (≈70%) ↓ Histamine (≈60%) with 200 µM	-	↓ IL-8, IL-13, TNF-α (dd)	↓ p-Lyn, p-PLCγ, p-IP3R ↓ p-38, p-IKK, NF-κB ↓ Lyn kinase activity	[33]
EPA/DHA	100 µM	LAD2	IgE-biotin/SA	↓ β-hex (25%)	↓ cys-LT (80%/76%)	-	↓ FcεRI localization into lipid rafts (> 50%) ↓ p-Lyn (37%/50%), p-Syk (33%/40%) ↓ p-LAT (37%/43%)	[34]
ALA, EPA, DHA	50, 75, 100 μM	BMMC, MC/9	IONO/PMA or Anti-DNP-IgE/DNP-BSA	-	-	↓ IL-4, IL-5, IL-13 (dd)	↓ GATA-1 and GATA-2	[35]
EPA, DHA	100 μM	HMC-1	IONO/PMA	↔ β-hex	↓ PGD_2_ (≈75%)	↓ IL-4 (≈70%), IL-13 (≈65%)	↓ p-ERK, p-JNK, p-p38	[36]
Butyrate	2 mM	BMMC	IgE/TNP-BSA	↔ β-hex	-	↓ TNF-α and IL-6 (≈70%)	↓ p-ERK, p-JNK, p-p38	[37]
Butyrate, Propionate	1, 5, 25 mM	BMMC PBMCMC	IgE/DNP-HSA or C48/80, SP	↓ β-hex (≈90% with 25 mM)	-	↓ IL-6 and TNF-α (≈90–100% with 25 mM)	↓ p-Btk (64%), p-Syk (43%), p-LAT (70%)	[38]
Glutamine/ Arginine	10 mM/2 mM	hiMC	Human myeloma IgE/anti-human IgE	↔ β-hex	↓ LTC_4_ (≈45%)	↓ CCL2, CCL4, CXCL8 and TNF-α (≈50%)	↓ p-ERK1/2, p-JNK, p-p38 ↓ p-MEK1/2 (≈30–45%), p-Akt (≈25%)	[39]
Glycine	250, 500 µg/mL	RBL-2H3	IgE/DNP-BSA	↔	-	↓ TNF-α (≈60%), IL-4 (≈75%), IL-13 (≈45%) with 250 µg/mL	-	[40]
Calcitriol	10 nM	BMMC, HMC-1, RBL-2H3, p815	DNP-IgE/DNP	↓ Histamine (≈90–100%)	-	↓ TNF-α (≈90–100%)	↓ p-Syk, p-p38, p-NF-κB(p50/65)	[41]
Calcidiol	10^−8^, 10^−7^, 10^−6^ M	BMMC	IgE/DNP-HSA	↓ Histamine (dd; 23–34%)	↓ cys-LT (dd; 34–44%)	↓ TNF-α and IL-6 (35% with 10^−8^, 10^−7^ M)	-	[27]
Calcitriol	10^−8^, 10^−7^ M	CBMC, PBMC	Human myeloma IgE/anti- human IgE	↓ Histamine (dd; ≈20, ≈30%)	↓ cys-LT (dd; ns, ≈20%)	↓ TNF-α (dd; ≈50, ≈50%) ↔ IL-10	-	[27]
Calcidiol	10^−8^, 10^−7^, 10^−6^ M	CBMC, PBMC	Human myeloma IgE/anti- human IgE	↓ Histamine (dd; ≈10, ≈30%)	↓ cys-LT (dd; ≈15–30%)	↓ TNF-α (dd; ≈10–30%) ↔ IL-10	-	[27]
Calcidiol	10^−9^–10^−6^ M	LAD2	IgE of sera of CSU patients	-	-	-	↓ p-Akt, p-p38, HIF-α, p-NF-κB	[42]
Carotenoids *	10 µM	RBL-2H3	Anti-DNP-IgE/DNP-BSA	↓ β-hex (≈70–30%)	-	-	-	[43]
Astaxanthin	100, 200, 400 µg/mL	RBL-2H3	PMA + calcium ionophore A23187	↓ β-hex, histamine (dd; ≈10–30%)	-	-	-	[44]
Kaempferol	100, 200 µM	LAD2	IgE/DNP-HSA, C48/80	↓ β-hex (≈60%, ≈80%) ↓ Histamine (≈50%, ≈50%)	-	↓ TNF-α (≈45%, ≈75%) ↓ IL-8 (≈55%, ≈80%) ↓ MCP-1 (≈20%, ≈50%)	↓ Lyn, Syk, Btk, Akt, MAPK, NF-κB ↓ PLCγ, IP3R, PKC, Ca^2+^	[45,46]
Kaempferol	10, 20 µM	RBL-2H3	Anti-DNP-IgE/DNP-BSA	↓ β-hex (≈50, ≈70%)	↓ PGD_2_ (≈20%) ↓ PGF2α (≈90%)	-	↓ p-Syk, p-PLCγ, p-PKCµ, p-ERK ↓ p-cPLA_2_, COX-2	[47]
Kaempferol	25–50 µM	BMMC	Anti-TNP-IgE/TNP-BSA	-	-	↓ TNF (≈80%), IL-6 (≈80%) and IL-13 (≈86%) with 50 µM	↓ PLCγ, FcεRI surface expression ↑ SHIP1	[48]
Quercetin	100, 200 µM	LAD2	IgE/DNP-HSA, C48/80	↓ β-hex (≈70%, ≈60%) ↓ Histamine (≈80%, ≈60%)	-	↓ TNF-α (≈40%), IL-8 (≈70%, ≈90%) ↓ MCP-1 (≈90%, ≈50%), IL-13 (≈50%)	↓ p-Lyn, p-PLCγ, p-IP3R, p-ERK1/2 ↓ p-IKK, NF-κB	[49,50]
ARE	20 µM	RBL-2H3	IgE/DNP-BSA	↓ Histamine (≈40%)	↓ PGE_2_ (≈90%) ↓ LTB_4_ (≈50%)	↓ TNF-α (≈40%), IL-4 (≈20%)	↓ p-Syk, p-PLCγ, p-PKCµ ↓ p-ERK, p-JNK, p-p38, COX-2, 5-LO	[51]
Myricetin	100 µM	LAD2	Streptavidin	↓ β-hex (≈50%)	-	↓ TNF-α (≈60%), IL-8 (≈70%) ↓ MCP-1 (≈40%)	↓ p-PLCγ1 (≈90%), p-Akt (≈70%) ↓ p-NF-κB (≈60%), p-p38 (≈45%)	[52]
Myricetin	10, 20, 40 µM	RBL-2H3	Anti-DNP-IgE/DNP-BSA	↓ β-hex (≈60%, ≈40%, ≈20%)	-	↓ IL-4 and TNF-α (≈60% with 20 µM)	↓ p-Syk, p-PLCγ ↓ IκBα, NF-κB(p65, p50)	[53]
Luteolin	20 µM	LAD2	IgE/DNP-BSA, C48/80	↓ β-hex (≈80%) ↓ Histamine (≈60%)	↓ PGD_2_ (≈40%)	↓ TNF-α, MCP-1, IL-8, IL-13 (dd)	↓ PLCγ ↔ Lyn, Btk	[54]
Luteolin	5, 10, 20 µM	HMC-1 RPMC	PMA + calcium ionophore A23187 or C48/80	↓ Histamine (≈70% with 20 µM in RPBMC)	-	↓ IL-1β (dd; 27.3–81.2%) in HMC-1 ↓ TNF-α (dd; 31.9–76.8%) in HMC-1	-	[55]
Luteolin	5, 10, 20 µM	HMC-1.2	IL-33	-	-	↓ IL-31 (dd; ≈17–70%)	↓ p-ERK, p-JNK, p-p38 ↓ p-p65, p-PKC, p-IKK	[56]
EGCG	0.1, 1, 10 µg/mL	HMC-1	RANKL	↓ Histamine (dd; ≈20–40%)	-	↓ TSLP (≈45%), IL-1β (≈80%), IL-6 (≈20%), IL-8 (≈80%) with 10 µg/mL	↓ p-PI3K, p-Akt, p-IκBα ↓ p-ERK, p-p38, p-JNK	[57]
EGCG″Me + eriodityol/hesperetin	1, 5, 25 µM	RBL-2H3	Anti-DNP-IgE/DNP-HSA	↓ β-hex (dd)	-	-	↓ 67LR/sGc/ASM	[58,59]
Naringenin	10 µM	HMC-1	TSLP	-	-	↓ TNF-α (≈70%), IL-13 (≈40%)	↓ p-STAT6 and MDM2 ↑ p53 and PARP	[60]
Nobiletin/ Tangeretin	15, 45, 100 µM	hiMC	LPS/sCD14	-	-	↓ CXCL8, CCL3, CCL4, IL-1β (dd)	↓ NF-κB	[61]
Nobiletin/ Tangeretin	15, 45, 100 µM	hiMC	Human myeloma IgE/anti-human IgE	Nobiletin: ↓ β-hex (dd; ≈20–40%)	Nobiletin: ↓ LTC_4_	↓ CXCL8, CCL3, CCL4, TNF-α (dd) Tangeretin: ↓ IL-1β	↓ p-ERK1/2	[61]
Nobiletin/ Tangeretin	10, 25 µM	RBL-2H3	PMA or histamine	-	-	↓ IL-4 (≈50%, ≈60%) and TNF-α (≈45%, ≈50%) with 25 µM	↓ NF-κB(p65), p-c-Jun, p-p38 ↓ PKC	[62]
Resveratrol	50, 100 µM	hiMC	mAb 22E7 (IgE-dependent activation)	↓ β-hex (≈75% at 50 µM)	-	↓ CXCL8, CCL2, CCL4, TNF-α, CCL3 (dd; ≈80–100%)	↓ p-STAT3 and p-ERK1/2 in nuclear and mitochondrial fractions	[16]
Resveratrol	100 µM	hsMC	IgE/NP-BSA	↓ β-hex (≈80%)	↓ PGD_2_	↓ TNF-α, IL-6	↓ p-Akt, p-p38, COX-2; ↔ Syk	[63]
Resveratrol	1–25 µM	BMMC	IL-33 and Anti-DNP-IgE/anti-IgE	↓ CD63 counts (≈70% with 25 µM)	-	↓ IL-6, IL-13, TNF-α (dd; ≈20–30% with 10 µM, ≈40–50% with 25 µM)	↓ p-Akt ↔ p-IKKα/ß, p-p65, p-p38, p-MK2	[64]
Resveratrol	10 µM	BMMC	Anti-DNP-IgE/DNP-HSA	↓ β-hex (≈65%)	↓ LTC_4_ and PGD_2_ (≈80%)	↓ IL-6 and TNF-α (≈70%)	↓ p-Akt, p-p38, p-Syk, p-PTP1B	[65]
Resveratrol	10 µM	RBL-2H3	IL-33 and IgE/DNP-HSA	-	-	↓ IL-6, IL-13, TNF-α, MCP-1	↓ p-p38, IκBα, NF-κB (p65) ↔ ST2, p-ERK1/2, p-JNK	[66]
Resveratrol	1–25 µM	RBL-2H3	Anti-DNP/DNP-HSA	-	-	↓ TNF-α, IL-4, IL-3, IL-13 (dd)	↓ p-p38, p-ERK1/2, p-JNK, p-Src	[67]
Resveratrol	50, 100, 200 µM	LAD2	C48/80	↓ β-hex (dd: ≈20–90%) ↓ Histamine (dd; ≈20–80%)	↓ PGD_2_	↓ MCP-1 (≈40%), TNF-α (≈60%) ↓ IL-1β (≈80%), TNF-α, IL-8 (dd)	↑ Nrf2, Ho-1, Nqo-1 (≈50–100%) ↓ MRGPRX2 mRNA expression	[68]
Resveratrol	0.03, 0.3, 3 µM	HMC-1	PMA + calcium ionophore A23187	-	-	↓ TSLP (≈25% with 3 µM)	↓ RIP2, caspase-1 ↓ NF-κB, p-IκBα	[69]
Curcumin	5–30 µM	RBL-2H3	Anti-DNP-IgE/DNP-BSA	↓ β-hex (dd; ≈50–80%) ↓ Histamine (dd; ≈30–60%)	-	-	↓ PKC-δ translocation	[70]
Curcumin	10 µM	BMMC	Anti-DNP-IgE/DNP-HSA	-	↓ LTC_4_ and PGD_2_ (≈80%)	-	↓ p-Akt, p-IKK, p-p65, p-MAPK ↓ p-PLCγ, p-cPLA_2_, 5-LO	[71]
Curcumin	10 µM	HMC-1	OVA or PMA	↓ Histamine (≈70%) with OVA	-	↓ TNF-α (≈60%), IL-1β (≈70%), IL-6 (≈70%), IL-8 (≈70%) with PMA	↓ p-ERK1/2, p-p38, p-JNK ↓ p-IκBα, NF-κB(p65)	[72]
BDMC	25, 50 µM	HMC-1	PMA + calcium ionophore A23187	-	-	↓ IL-6 (≈60%), IL-8 (≈90%), TNF-α (≈60%) with 50 µM	↓ p-ERK, p-p38, p-JNK ↓ NF-κB, IκBα	[73]
CE	0.1, 1, 10 µM	hiMC RBL-2H3	IgE/anti-human IgE IgE/DNP	↓ β-hex (dd; ≈50–80%)	↓ cys-LT (dd)	↓ CXCL8, CCL2, CCL3, CCL4, TNF-α (dd; ≈50–95%)	↓ p-Akt, p-ERK, p-JNK, p-p38	[74]
CA	100, 250, 500 µM	hiMC RBL-2H3	IgE/anti-human IgE IgE/DNP	↓ β-hex (≈70–90%)	↓ LTC_4_ (> 90%)	↓ CXCL8 (dd) ↓ CCL2, CCL3, CCL4 (dd)	↓ p-ERK ↓ p-PLCγ1	[75]
4-chloro-CA 4-trifluoro-CA	40, 50, 60 µM	RBL-2H3	PMA + calcium ionophore A23187	↓ β-hex (dd; ≈40–60%)	-	↓ IL-4, TNF-α (dd)	↓ p-MEK1/2, p-MKK4 ↓ p-ERK, p-p38, p-JNK, COX-2	[76]

* Fucoxanthin, zeaxanthin, β-carotene, 3-hydroxyechinenone, astaxanthin, fucoxanthinol, lycopene, β-cryptoxanthin, and siphonaxanthin. Abbreviations: β-hex, β-hexosaminidase; 5-LO, 5-lipoxygenase; 67LRs, 67-kDa Laminin Receptor; Akt, protein kinase B; ARE, Aceriphyllum rossii; ASM, acid sphingomyelinase; BDMC, bisdemethoxycurcumin; BMMC, bone marrow-derived mast cell; BSA, bovine serum albumin; Btk, bruton’s tyrosine kinase; C48/80, compound 48/80; CA, cinnamaldehyde; CCL, CC-chemokine ligand; CBMC, cord blood-derived mast cell; CE, cinnamon extract; COX, cyclooxygenase; cPLA_2_, cytosolic phospholipase A_2_; CXCL, C-X-C motif ligand; cys-LT, cysteinyl leukotriene; dd, dose-dependent; DNP, dinitrophenol; ERK, extracellular signal-regulated kinase; FcεRI, high affinity IgE receptor; hiMC, human intestinal mast cells; HMC-1, human mast cell line 1; HO-1, heme oxygenase 1; HSA, human serum albumin; hsMC, human skin mast cells; IKK, inhibitor of nuclear factor kappa-B kinase; IONO, ionomycin; IP3R, inositol trisphosphate receptor; IκBα, inhibitor of nuclear factor kappa B; JNK, c-Jun N-terminal kinase; LAD2, laboratory of allergic diseases 2; LAT, linker for activation of T cells; LPS, lipopolysaccharide; LT(C_4_; B_4_), leukotriene (C_4_; B_4_), mAb, monoclonal antibody; MAPK, mitogen-activated protein kinase; MCP-1, monocyte chemoattractant protein-1; MDM2, murine double minute 2; MEK, MKK, mitogen-activated protein kinase kinase; mMCP-1, mucosal mast cell protease-1; MRGPRX2, Mas related G protein-coupled receptor type 2; NF-κB, nuclear factor kappa B; NP, 4-hydroxy-3-nitrophenylacetyl; Nqo-1, NAD(P)H quinone oxidoreductase-1; Nrf2, nuclear factor erythroid 2-related factor 2; ns, not significant; OVA; ovalbumin; p, phospho; p38, p38 mitogen-activated protein kinase; p50,p65, nuclear factor kappa B subunit p50, p65; PARP, poly(ADP-ribose)polymerase; PBMCMC, peripheral blood mononuclear cell-derived mast cell; PG (D_2_, E_2_, F2α), prostaglandin (D_2_; E_2_; F2α), PI3K, phosphoinositide 3-kinase; PKC, protein kinase C; PLCγ1, phospholipase Cγ 1; PMA, phorbol-12-myristate-13-acetate; PTP1B, protein-tyrosine phosphatase 1B; RANKL, receptor activator of nuclear factor kappa-Β ligand; RBL-2H3, rat basophilic leukemia cell; RPMC, rat peritoneal mast cell; SA, streptavidin; SHIP-1, Src homology 2 (SH2)-containing inositol phosphatase 1; sGC, soluble guanylyl cyclase; SP, substance P; ST2, interleukin 1 receptor-like 1; STAT, signal transducer and activator of transcription; TNP, trinitrophenol; TSLP, thymic stromal lymphopoietin; CXCL8/IL-8, MCP-1/CCL2 as listed in the literature; ↓, decrease in %; ↑, increase in %; ↔ no effect; ≈given values are approximated.

**Table 2:** Overview of the effects of dietary components on MC in vivo.

Substance	Dosage	Animal Model	Allergy Model (Trigger)	Prestored MC Mediators	De Novo Synthesized MC Mediators	Signaling Molecules	Others	Ref.
ALA	1, 2, 4 mg/kg (in 0.2 mL saline) (i.v. into tail vein)	♂ C57BL/6 mice	PCA (C48/80 or SP, i.d.)	↓ Serum histamine (dd; ≈20–60%)	↓ IL-8 (≈55%), IL-13 (≈45%) and TNF-α (≈70%) with 4 mg/kg	-	↓ Hind paw thickness, vasodilation ↓ Eosinophils ↓ Degranulated MC	[33]
Fish oil *	40 mg/kg (oral, daily for 6 wk)	♂ NC/Nga mice	AD (TNCB, dorsal skin)	-	-	↓ GATA-1 (≈40%)	↓ Infiltration of MC, eosinophils ↓ MC count	[35]
Sodium butyrate	450 mg/kg (oral, daily for 2 wk)	Weaned piglets	-	↓ Histamine (≈40%) in jejunum mucosa	↓ IL-6 (≈30%), TNF-α (≈45%) ↓ IL-13 (≈20%) in jejunum mucosa	↓ p-JNK/JNK ↔ p-ERK, p-p38	↓ MC tryptase (≈40%), MC count (≈20%) ↓ Degranulated MC (≈50%)	[77]
L-Glutamine	85 mM (duodenal infusion cannula over 2–3 min)	♂ Sprague Dawley rats	Dietary fat	↑ Lymphatic histamine (≈60%)	↑ Lymphatic PGD_2_ (≈70%)	-	↑ RMCPII	[78]
Glycine	50, 100 mg (in 200 µL water) (oral, 1× wk for 5 wk)	C3H/HeOuJ mice	CMA (whey)		↓ mMCP-1 in serum and jejunum (≈30%)	-	↓ Serum whey specific IgE1 (≈10%, ≈20%)	[40]
Fucoxanthin	150 nmol by feeding needle or mouse ear	♂ ICR mice	AA, PMA, OXA	-	-	-	↓ Ear swelling (oral: 20–28%, percutane: >50%)	[79]
Astaxanthin	1, 2 mg/mL (in 100 µL) (ears and back skin, 3× wk for 4 wk)	HR-1 mice	AD (PA, ear dorsum)	-	↓ Serum TNF-α (≈50%, ≈60%) ↓ Serum IL-1β (≈30%, ≈50%) ↓ Serum IL-6 (≈90%, ≈80%)	↓ p-IκBα ↓ iNOS, COX-2	↓ MC count (≈50%, ≈70%) ↓ Serum IgE levels, MDA ↑ GSH, GPx-1, HO-1	[80,81]
Astaxanthin	1 mg/mL (in AOO) (ear dorsum, 1× every 2 days for 8 days)	♂ BALB/c mice	CD (DNFB, ear dorsum)	-	↓ TNF-α (≈60%) and IFN-γ (≈70%) in ear tissue	-	↓ Ear thickness and weight	[44]
Astaxanthin (free or liposomal)	1 mg/mL (in 200 µL AOO) (dorsal back skin, 3× wk for 4 wk)	♂ SKH-1 mice	AD (PA, dorsal skin)	-	↓ IL-1β, IL-6, IL-4, IL-13 in skin tissue (L-AST: 20–30% higher inhibition)	↓ p-STAT3, p-IκBα ↓ NF-κB(p50/p65) ↓ iNOS, COX-2	↑ GPx-1, HO-1 ↓ Serum IgE levels, MC count in skin ↓ Epidermal thickening	[82]
Astaxanthin	100 mg/kg (oral, 3× wk for 26 days)	♂ NC/Nga mice	AD (Mites)	-	↓ Eotaxin, MIF, IL-4, IL-5 ↔ TNF-α, IL-1β in skin tissue	-	↓ Serum IgE, eosinophils (≈80%) ↓ Degranulated and total MC count (≈50%) ↓ Spontaneous scratching behavior	[83]
Lycopene	0.1% (*w*/*w*) of standard chow diet (daily for 28 days)	♀ BALB/c mice	FA (OVA)	-	↓ IL-4 (≈60%), IL-13 (≈60%) ↓ IL-9 (≈70%), mMCP-1 (≈50%) ↔ IL-5 in colonic mucosa	-	↓ MC count (≈50%)	[84]
Kaempferol	5, 10, 20 mg/kg (in DMSO) (oral)	♂ C57BL/6 mice	PCA (OVA or C48/80, i.p./i.d.)	↓ Serum histamine (≈80% with OVA and 10 mg/kg) (≈60% with C48/80 and 20 mg/kg)	↓ Serum TNF-α and IL-8 (dd) ↓ Serum MCP-1 (dd)	-	↓ Hind paw swelling ↓ Primary MC activation from paw skin ↓ Rehabilitated the hypothermia	[45,46]
Quercetin	1, 2, 4 mg/mL (i.v. into each mouse front paw)	C57BL/6 mice	Pseudo allergy (C48/80 or SP, i.d.)	↓ Serum histamine (≈0%, ≈20%, ≈40%)	↓ MCP-1 (≈40%, ≈50%, ≈60%) ↓ IL-8 (≈40%, ≈40%, ≈50%) in serum	-	↓ Hind paw thickness, vasodilation ↓ Degranulated MC ↓ Eosinophils	[50]
Quercetin	1, 2, 4 mg/kg (oral, daily for 7 days)	♂ C57BL/6 mice	AC (OVA, i.p.)	↓ Serum histamine (≈20%, ≈60%, ≈60%)	↓ IL-4 (dd; ≈20–90%) ↓ TNF-α (dd; ≈10–40%) in serum	-	↓ Serum IgE, eosinophils ↓ Degranulated MC (≈50%) ↓ Vascular permeability	[49]
Myricetin	50 mg/kg (oral, daily for 4 days)	♂ BALB/c mice	ICU (DNFB, ear and back skin)	↓ Serum histamine (43%)	↓ IL-4 (51%), TNF-α (43%) and MCP-1 (67%) in serum	↓ PI3K, Akt ↓ NF-κB	↓ Serum IgE levels (45%) ↓ Degranulated MC (42%) ↓ Scratching behavior, ear swelling	[52]
Myricetin	12.5, 25, 50 mg/kg (oral)	♂ C57BL/6 mice	PCA (OVA)	↓ Serum histamine (75%) with 50 mg/kg	↓ IL-4 (47%), TNF-α (42%) and MCP-1 (52%) in serum with 50 mg/kg		↓ OVA-induced PCA reaction ↓ Degranulated MC	[52]
Luteolin	20 mg/kg (in PBS) (oral)	♂ ICR mice	(C48/80, i.d.)	-		-	↓ Scratching behavior (≈70%) ↓ Skin vascular permeability (≈60%)	[55]
EGCG	25, 50, 100 mg/kg (oral, 1× day for 10 days)	♀ BALB/c mice	AR (OVA, nasal vestibule)	↓ Serum histamine (dd; ≈10–30%)	↓ IL-1β (≈40%), IL-4 (≈50%) and IL-6 (≈60%) in nasal mucosa with 100 mg/kg	↓ COX-2 (≈50%)	↓ Nasal rubbing, sneezing ↓ Serum IgE levels (dd; ≈10–30%)	[85]
Naringenin	100 mg/kg (in 2 mL saline and CMC) (oral, 1× day for 7 days)	♀ Sprague Dawley rats	AR (OVA, i.p., nostrils)	-	↓ IL-4 (≈10%), IL-5 (≈20%) in plasma	-	↓ Nasal scratching and sneezing (≈60%) ↓ Desquamation in the nasal epithelium ↓ Serum IgE levels (≈30%)	[86]
Naringenin	50, 100 mg/kg (i.p., 1× day for 6 days)	♂ NC/Nga mice	AD (DNFB, ear and back skin)	-	↓ IFN-γ (≈45%, ≈35%) by activated lymph node CD4^+^ T cells	-	↓ CD4^+^, CD8^+^ cells ↓ Serum IgE levels (≈50%) ↓ Degranulated MC (≈40%) ↓ Ear swelling, back skin lesions	[87]
Nobiletin/ Tangeretin	25 mg/kg (in cremophor) (oral)	♂ ICR mice	(Histamine or C48/80, i.d.)	-	↓ TNF-α (94%/96%) and IL-4 (84%/96%) in skin tissue	↓ p-p65 ↓ p-c-Jun	↓ Scratching behavior (≈70% with histamine), ≈60% with C48/80) ↓ Vascular permeability (≈50%)	[62]
Resveratrol	10, 20 mg/kg (oral, 1× day for 13 days)	♀ BALB/c mice	FA (OVA, i.p., oral)	↓ Serum histamine (dd; ≈25% -50%)	↓ Serum mMCP-1 (dd; ≈30–50%)	-	↓ OVA-specific serum IgE (≈45%)	[88]
Resveratrol	50 mg/kg (in drinking water for 28 days)	♀ BALB/c mice	FA (OVA, i.p., oral)	-	-	↓ IL-3Rα mRNA (≈80%)	↓ MC numbers (≈60%)	[89]
Resveratrol	15 mg/kg (i.p., 1x day for 5 days)	♂ Sprague Dawley rats	IIR	↓ Intestinal β-hex (≈50%)	↓ TNF-α (≈50%), IL-1β (≈40%) ↓ IL-18 (≈50%)	↓ Mucosal NLRP3 and caspase-1 p20 (≈50%), IL-1β p17 and IL-18 (≈60%)	-	[90]
Resveratrol	5 mg/kg (i.p., 1× day for 7 days)	♂ Sprague Dawley rats	(IL-33, i.p.)	-	↓ Plasma IL-6 (≈50%), IL-13 (≈40%), TNF-α (≈60%), MCP-1 (≈50%)	-	-	[66]
Resveratrol	10 mg/kg in 100 µL (oral, once)	♂ BALB/c mice	PCA (anti-DNP-IgE/DNP-HSA, i.d./i.v.)	↓ Plasma histamine (≈50%)	↓ MCP-1 (≈50%), MIP-2 (≈40%) in dorsal dermis	↓ Syk, ↓ PLCγ, PKCµ	↓ Vascular permeability (≈75%), thickness of ears (≈50%) ↓ Degranulated MC in dorsal dermis	[67]
Resveratrol	5, 10, and 20 mg/kg (i.g.)	♂ C57BL/6 mice	Pseudo allergy (C48/80, i.v.)	↓ Serum histamine (≈60%, ≈60%, ≈70%)	↓ MCP-1 (≈50%, ≈70%, ≈80%) ↓ TNF-α (≈30%, ≈50%, ≈70%) ↓ IL-8 (≈20%, ≈30%, ≈50%)	-	↓ Degranulated MC (ns; ≈50%; ≈70%) ↓ Paw thickness (≈20%; ≈60%; ≈60%)	[68]
Resveratrol	2.5 µg/mL (patches on back skin, 1× day for 7 days)	♀ C57BL/6J	AD (OVA, patches)	-	↓ CCL2, CCL3 and CCL5 in skin tissue	↓ p-SphK1 ↓ p-STAT3 ↓ p-NF-κB(p65)	↓ FcεRIα mRNA expression ↓ Epidermal thickening ↓ Skin MC activation	[91]
Curcumin	25, 50 mg/kg (oral)	ICR mice	PSA (IgE/DNP-HSA, i.v.)	↓ Serum histamine (≈30%, ≈40%)	↓ Serum LTC_4_ (≈30%, ≈50%) ↓ Serum PGD_2_ (≈20%, ≈50%)	-	-	[71]
Curcumin	100, 200 mg/kg (oral, 1× day for 3 days)	♀ BALB/c mice	AR (OVA, i.p./i.n.)	↓ Serum histamine (≈50%, ≈70%)	↓ Serum TNF-α (≈60%, ≈70%)	↓ Fyn, Lyn, Syk	↓ OVA-sIgE (≈70%, ≈80%) ↓ Nasal sneezing and rubbing	[72]
BDMC	100, 200 mg/kg (oral, 1× day for 10 days)	♀ BALB/c mice	FA (OVA, i.g., nasal vestibule)	↓ Serum histamine (≈20%, ≈50%)	↓ Serum IL-4, IL-5 and IL-13 (dd; < 30%) ↓ Serum mMCP-1, ↑ IFN-γ	↓ GATA-3, ↓ NF-κB(p65) ↓ p38, JNK, ERK	↓ OVA-sIGE (≈20%, ≈50%) ↓ OVA-sIgG1 (≈20%, ≈50%) ↓ Diarrhea, anaphylaxis symptoms	[92]
BDMC	100, 200 mg/kg (oral, 1× day for 10 days)	♂ BALB/c mice	AR (OVA, i.p.)	↓ Serum histamine (≈30%, ≈40%)	-	-	↓ OVA-sIgE levels (≈30%, ≈40%) ↓ Nasal rubbing	[93]
Cinnamon extract	4.5 mL/kg (in tap water for 6 wk)	C57BL/6J mice	-	-	↔ IL-4 and IL-1β	-	↓ Carboxypeptidase A ↓ MC tryptase	[74]

* containing 20–31% omega-3 fatty acids. Abbreviations: ♀, female; ♂, male; β-hex, β-hexosaminidase; AA, arachidonic acid; AC, allergic conjunctivitis; AD, atopic dermatitis; Akt, protein kinase B; ALA, alpha-linolenic acid; AOO, acetone: olive oil (4:1) solution; AR, allergic rhinitis; BDMC, bisdemethoxycurcumin; C48/80, compound 48/80; CCL, chemokine (C-C motif); CD, contact dermatitis; c-Jun, proto-oncogene c-Jun; CMA, cow’s milk allergy; COX, cyclooxygenase; dd, dose-dependent; DMSO, dimethyl sulfoxide; DNFB, 1-Fluoro-2,4-dinitrobenzene; DNP, dinitrophenol; EGCG, epigallocatechin gallate; ERK, extracellular signal-regulated kinase; FA, food allergy; Fyn, Fyn tyrosine kinase; GATA, GATA-binding protein; GPx-1, glutathione peroxidase 1; GSH, glutathione; HO-1, heme oxygenase-1; HSA, human serum albumin; i.d., intradermal; i.g., intragastric; i.n., intranasal; i.p., intraperitoneal; i.r., intrarectal; i.v., intravenous; ICU, immunologic contact urticaria; IFN-γ, interferon gamma; IIR, intestinal ischemia-reperfusion; iNOS, inducible nitric oxide synthase; IκBα, inhibitor of kappa B alpha; JNK, c-Jun N-terminal kinase; LTC_4_, leukotriene C_4_; Lyn, Lyn tyrosine kinase; MCP-1, monocyte chemoattractant protein-1; MDA, malondialdehyde; MIF, macrophage migration inhibitory factor; MIP-2, macrophage inflammatory protein-2; mMCP-1, mucosal mast cell protease-1; NF-κB, nuclear factor kappa B; NLRP3, NOD-, LRR- and pyrin domain-containing protein 3; OVA, ovalbumin; OXA, oxazolone; PMA, phorbol-12-myristate-13-acetate; p, phospho; p38, p38 mitogen-activated protein kinase; PA, phthalic acid; PCA, passive cutaneous anaphylaxis; PGD_2_, prostaglandin D_2_; PI3K, phosphoinositide 3-kinase; PKC, protein kinase C; PLCγ, phospholipase C γ; PSA, passive systemic anaphylaxis; RMCPII, rat mucosal mast cell protease II; ROS, reactive oxygen species; SP, substance P; SphK1, sphingosine kinase 1; STAT3, signal transducer and activator of transcription 3; TNCB, 2,4,6-trinitrochlorobenzene; UC, ulcerative colitis; wk, week; CXCL8/IL-8, MCP-1/CCL2 as listed in the literature; ↓, decrease in %; ↑, increase in %; ↔ no effect; ≈ given values are approximated.

**Table 3:** Overview of the effects of dietary components on allergic rhinitis in RCT.

Substance	Dosage	Study Type and Duration	Study Population	Results	Ref.
Vitamin D	Vitamin D3 1.5 × 10^6^ IU nasal drops (1× wk) + desloratadine citrate disodium (8.8 mg/day)	RCT (1 month)	*N* = 60 children/adults with mild seasonal pollen AR Mean age: 27.3	↑ Serum 25(OH)D_3_ level (≈50%) ↓ AR symptoms score (≈40%) ↓ Serum IL-4 (≈20%) ↓ Peripheral blood eosinophils (≈20%)	[136]
Vitamin D	Vitamin D 50,000 IU (1× wk, oral) + cetirizine	RCT (2 months)	*N* = 68 adults with vitamin D deficiency and AR Mean age: 29.4 years	↑ Serum 25(OH)D_3_ level (≈60%) ↓ AR symptom severity (≈24%) ↓ Rhinorrhea (≈26%), nasal itching (≈27%), sneezing (≈28%) and postnasal drip (≈28%)	[137]
Quercetin	200 mg/day (4 × 50 mg tablets/day divided into two doses)	RCT (1 month)	*N* = 60 adults with AR Mean age: 46.85 years	↓ Total AR symptom score (≈27%) ↓ Sleeping disorder (≈40%) ↓ Itching sensation other than ocular/nasal itching sensation ↓ Eosinophil count in nasal discharge	[138]
*O*- methylated EGCG	Benifuuki green tea (700 mL/day) (5.83 mg/100 mL *O*-methylated EGCG)	RCT (4 months)	*N* = 26 adults with Japanese cedar pollinosis Mean age: 39.6 years	↓ Runny nose (≈8%), itchy eyes (≈18%) and tearing (≈17%) ↓ Total nasal symptom score (≈10%) and total ocular symptom score (≈17%) ↓ Nasal symptom-medication score (≈8%) ↓ Ocular symptom-medication score (≈12%) ↓ Peripheral eosinophil counts (≈30%)	[139]
Resveratrol	Resveratrol (0.1%) (2 sprays (100 µL/spray) in each nostril 3× day)	RCT (1 month)	*N* = 151 adults with severe persistent AR Age: 18–60 years	↓ Nasal symptoms ↓ Blood levels of IgE (≈40%), IL-4 (≈30%), and TNF-α (≈10%) ↓ Eosinophile number in blood (≈80%)	[140]
Resveratrol	Resveratrol (0.05%) + carboxymethyl-β-glucan (0.33%) (2 sprays (100 µL/spray) in each nostril 3×/day)	RCT (2 months)	*N* = 68 children with AR Mean age: 7.9 years	↓ Itching, sneezing, rhinorrhea, and obstruction ↓ Antihistamine use	[141]
Curcumin	500 mg in capsules (1× day)	RCT (2 months)	*N* = 241 adults with perennial AR Mean age: 32.6 years	↓ Nasal symptoms (≈60%) ↓ Sneezing (≈60%), itching (≈60%), rhinorrhea (≈80%), obstruction (≈50%) ↑ Nasal airflow baseline ↓ IL-4 (≈30%), TNF-α (≈40%) and ↑ IL-10 (≈20%) in activated MNC ↓ IL-8 (≈30%) and ↑ sICAM-1 (≈30%) in PMN ↔ IFN-γ and IL-17 in MNCs, PGE_2_ and LTC_4_ in PMN	[142]
Cinnamon bark extract	IND02 100 µg in 100 µL (2 sprays (100 µL/spray) in each nostril 2× day)	RCT (7 days)	*N* = 60 adults with acute symptoms of AR Mean age: 43.25 years	↓ Nasal (47.7%) and eye (50.9%) symptoms, non-nose/eye symptoms (50.5%)↓ Activity limitation (41.8%) ↓ Sleep disorder (46%) ↓ Total white blood cells (≈20%) ↓ Neutrophils (≈20%)	[143]

Abbreviations: AR, allergic rhinitis; IFN-γ, interferon gamma; IgE, immunoglobulin E; IL, interleukin; IND02, cinnamon (cinnamomum zeylanicum) bark extract; LTC_4_, leukotriene C_4_; MNCs, mononuclear cells; PGE_2_, prostaglandin E_2_; PMNs, polymorphonuclear neutrophils; RCT, double-blind randomized controlled trial; sICAM-1, soluble intercellular adhesion molecule 1; TNF-α, tumor necrosis factor alpha; wk, week. ↓, decrease in %; ↑, increase in %; ↔ no effect; ≈ given values are approximated.

## Abbreviations

Akt	Protein kinase B
AR	Allergic rhinitis
BMMC	Bone marrow-derived mast cell
Btk	Bruton’s tyrosine kinase
C48/80	Compound 48/80
CBMC	Cord blood-derived mast cell
CCL	CC chemokine ligand
CXCL	C-X-C motif chemokine ligand
cys-LT	Cysteinyl-leukotriene
EGCG	Epigallocatechin gallate
ERK	Extracellular signal-regulated kinase
FcεRI	High affinity IgE receptor
hiMC	Human intestinal mast cell
HMC-1	Human mast cell line 1
hsMC	Human skin mast cell
JNK	c-Jun *N*-terminal kinase
LAD2	Laboratory of Allergic Disease 2
LTC_4_	Leukotriene C_4_
Lyn	Lck/Yes-related novel protein tyrosine kinase
MAPK	Mitogen-activated protein kinase
MCP-1/CCL2	Monocyte chemoattractant protein-1
mMCP-1	Mucosal mast cell protease-1
NF-κB	Nuclear factor kappa B
OVA	Ovalbumin
PBMCMC	Peripheral blood mononuclear cell-derived mast cell
RBL-2H3	Rat basophilic leukemia 2H3
RCT	Randomized controlled trial
Syk	Spleen tyrosine kinase
